# Cancer-Derived Exosomes as Effectors of Key Inflammation-Related Players

**DOI:** 10.3389/fimmu.2019.02103

**Published:** 2019-09-04

**Authors:** Norahayu Othman, Rahman Jamal, Nadiah Abu

**Affiliations:** UKM Medical Centre, UKM Medical Molecular Biology Institute, Kuala Lumpur, Malaysia

**Keywords:** Exosomes, cancer, inflammation, immune molecules, tumorigenesis

## Abstract

Exosomes, a category of small lipid bilayer extracellular vesicles that are naturally secreted by many cells (both healthy and diseased), carry cargo made up of proteins, lipids, DNAs, and RNAs; all of which are functional when transferred to their recipient cells. Numerous studies have demonstrated the powerful role that exosomes play in the mediation of cell-to-cell communication to induce a pro-tumoral environment to encourage tumor progression and survival. Recently, considerable interest has developed in regard to the role that exosomes play in immunity; with studies demonstrating the ability of exosomes to either metabolically alter immune players such as dendritic cells, T cells, macrophages, and natural killer cells. In this review, we summarize the recent literature on the function of exosomes in regulating a key process that has long been associated with the progression of cancer—inflammation and immunity.

## Introduction

### Inflammation and Cancer

The inflammatory response is a mechanism that is activated in response to tissue damage or recognition of pathogens, and is facilitated by the action of various cells and soluble mediators of the innate and adaptive immune system (1, 2). In the normal physiological context, once the homeostatic state has been achieved, through the elimination of the foreign pathogen or successful tissue repair, inflammation will be resolved (3). However, if inflammation becomes unregulated and is inadequately resolved it can result in chronic inflammation, which has been linked with an increase in the risk of malignant cell transformation and cancer (2, 4).

The inflammatory and the carcinogenic process share several molecular targets and signaling pathways including apoptosis, proliferation, and angiogenesis. Therefore, sustained exposure to the inflammatory process can contribute to the initiation, promotion, growth, and invasion of tumors by providing bioactive inflammation-related molecules to cells that can infiltrate the tumor microenvironment (1, 2). These molecules include integral players of cancer-related inflammation such as transcription factors NFκB and signal transducer activator of transcription 3 (Stat3), as well as primary inflammatory cytokines such as interleukin (IL)-1β, IL-6, and tumor necrosis factor alpha (TNF-α) (5–8). Accumulation of these inflammatory mediators leads to local and systemic immunosuppression associated with the progression of cancer. Furthermore, several reports have provided accumulating evidence that inflammatory factors are able to down-regulate DNA repair pathways and cell cycle checkpoints, which results in the buildup of random genetic alterations due to the destabilization of the cancer cell genome (9, 10). Additionally, cytokines such as TNF-α and IL-6 have also been reported to induce the production of free radicals leading to DNA damage and mutations that can further contribute to the initiation of tumors (11, 12).

While inflammation plays a significant role in the promotion of tumor progression, the recruitment of inflammatory cells and up-regulation of anti-inflammatory cytokines by the host is also aimed at suppressing tumor growth. For example, classically activated M1 macrophages exhibit anti-tumor activity and elicit anti-tumor adaptive immunity in the early stages of carcinogenesis (13), by damaging vascular cells and activating coagulation to induce hemorrhagic necrosis (14). However, in established and advanced neoplasia M2-polarized macrophages outweigh M1 macrophages and tumor cells will escape immune attack (13). The balance between the anti-tumor and pro-tumor properties of macrophages is demonstrated to be regulated by NF-κB, thus NF-κB can be targeted to favor the anti-tumor macrophage function (15, 16).

### Cancer-Derived Exosomes

**Figure 1:**
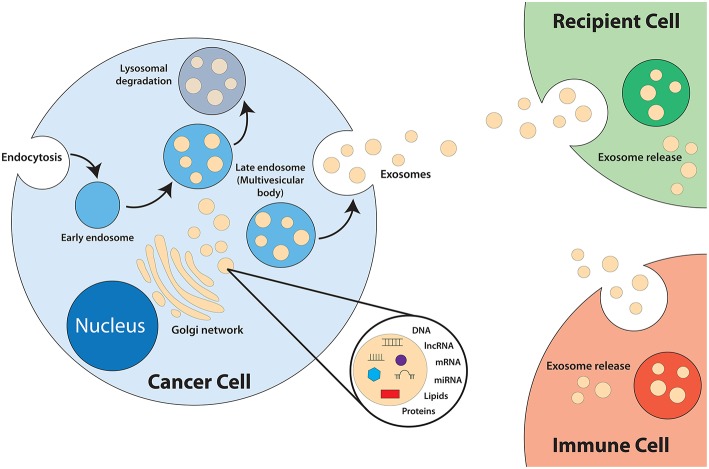
Biogenesis of exosomes.

Exosomes are a category of small lipid bilayer extracellular vesicles that measures approximately 30–100 nm (17–19). The process of exosomes biogenesis (Figure 1) begins with invagination of the plasma membrane to form endosomes. As the endosomes mature, inward budding of the endosome membrane will result in the formation of numerous intraluminal vesicles (ILV) that contain components of the cytosol, including nucleic acids and functional proteins (18, 20–24). The late endosome, now termed multivesicular bodies (MVB), will fuse with the plasma membrane to release the ILVs into the extracellular space, exosomes (25). Exosomes play an integral role in intercellular communication and act as shuttles by transmitting signals and transferring of their contents, thus playing a role in the regulation of physiological and pathological processes of diseases (26–28). The cargo of exosomes is made up of proteins, lipids, DNAs (mtDNA, ssDNA, dsDNA), and RNAs (mRNA, miRNA, long non-coding RNA), which are all functional when transferred into recipient cells (29–32). Exosomes are released from most cell types and can be found in all bodily fluids including urine, plasma, saliva, cerebrospinal fluid, amniotic fluid, and breast milk (33–36).

Extensive reports have illustrated that exosomes derived from tumors are significantly involved in the modulation of the biological activities of their recipient cells via the transfer of their oncogenic content. For example, exosomes play a role in the induction of normal cell transformation. A study has demonstrated that prostate cancer cell-derived exosomes are involved in the clonal expansion of tumors through reprogramming of adipose-derived stem cells via trafficking of oncogenic factors such as H-ras and K-ras transcripts, as well as oncogenic miRNAs miR-125b, miR-130b, and miR-155 (37). Breast cancer cell-derived exosomes have also been shown to promote normal cell transformation. It was determined that exosomes derived from cells and sera of breast cancer patients was able to promote the formation of tumors from non-tumorigenic epithelial cells in a Dicer-dependent manner (38). Other biological activities affected by tumor-derived exosomes include cell proliferation; as observed in colon cancer cells whereby a transfer of exosomes from colon tumor cells was able to induce a rise in cell proliferation and chemoresistance in acceptor cells both *in vitro* and *in vivo* (39). Up-regulation of angiogenesis has also been detected to be influenced by tumor-derived exosomes. One study demonstrated that leukemia cells-derived exosomes was able to transport miRNAs (miR-92a) to endothelial cells to modulate endothelial migration and tube formation (40). The metastatic ability of cells can also be modulated by tumor derived-exosomes. Exosomes isolated from melanoma cells of stage V patients is able to stimulate the formation of a metastatic niche by encouraging bone marrow-derived cells toward a pro-metastatic phenotype via up-regulation of the MET oncoprotein (41). Furthermore, there is potential for the development of exosomes as diagnostics biomarkers as they contain all the bioactive molecules of the cells they are derived from (42–46).

Recently considerable interest has developed in regard to the role that exosomes play in the immune response, with studies demonstrating the ability of exosomes to either promote or inhibit immune responses. For example, exosomes derived from thrombin-activated platelets are able to stimulate the proliferation, survival, and chemotaxis of hematopoietic cells (47) as well as activate monocytes to release pro-inflammatory cytokines and induce the activation of B cells (48). Furthermore, exosomes released by antigen presenting cells such as B-lymphocytes and dendritic cells contain MHC class I molecules that are able to potentially mediate antigen-specific T cell cross-priming (49–53). Natural killer (NK) cells-derived exosomes have also been shown to mediate anti-tumor activities via its cellular content perforin and granzyme B, which has cytotoxic activity against various tumor cell lines (54). It has also been reported that peptides expressed in exosomes derived from mast cells can be presented to dendritic cells to stimulate specific immune responses (55). Various immune suppressive roles of exosomes include the promotion of apoptosis in activated T cells via expression of death ligands FasL (56) and TRAIL (57); impairment of dendritic cell differentiation from monocytes (58); as well as the suppression of NK cells mediated cytotoxic responses (59).

## Pro-inflammatory Effects of Exosomes

### Monocytes

Monocytes are a subset of mononuclear leukocytes, or circulating white blood cells, which differentiates into macrophages and dendritic cells following stimulation by cytokines and other molecules (60, 61). Monocytes play a significant role in both innate and adaptive immunity (62) through the production of various effector molecules such as inflammatory cytokines, myeloperoxidase, and superoxide, to contribute toward and initiate local and systemic inflammation (63, 64). During chronic inflammation, cytokines have been reported to promote the creation of ideal growth conditions within the tumor microenvironment as well as to promote the progression of tumor growth (65–67). Tumor cells and its associated microenvironment can produce molecules that alter the recruitment, migration, differentiation, and functional properties of monocytes (68).

Exosomes produced and released by tumor cells contain various biomolecules that can be transmitted to recipient cells, including monocytes, to regulate their behavior. In a study conducted in 2013 by Bretz et al. it was demonstrated that exosomes obtained from malignant ascites of ovarian cancer patients were able to modulate the biological functions of monocytic cells (69). Results indicated that tumor-derived exosomes were transferred into the THP-1 human monocytic cells and significantly induced the production and secretion of various pro-inflammatory cytokines including interleukin (IL)-6, IL-1β, and IL-8, and tumor necrosis factor (TNF)-α, via TLR2 and TLR4 binding on the cell surface of monocytes, which subsequently activated nuclear factor κB (NFκB) and signal transducer and activator of transcription 3 (STAT3) (69). NF-κB is a significant regulator of inflammation, and constitutive activation of NFκB is often observed in cancer cells and is associated with an aggressive phenotype, which includes tissue invasion and metastasis as well as resistance to growth inhibition (70). NF-κB is activated downstream of the TLR-MyD88 pathway and inflammatory cytokines TNF-α and IL-1β (9). Furthermore it has recently been shown that STAT3 is required for the activation of NFκB and interaction between these two transcription factors is essential for the regulation of communication between inflammatory and cancer cells. Together, NFκB and STAT3 have the capability to regulate apoptosis, angiogenesis, and tumor invasion thus enabling resistance toward immune surveillance (1, 71).

The role of tumor-derived exosomes in the cross-talk with monocytes was also evaluated in a separate study focused on chronic lymphocytic leukemia (CLL). CLL-derived exosomes was found to play a role in the skewing of monocytes and macrophages toward a pro-tumorigenic phenotype with the release of tumor-supportive cytokines as well as the expression of immunosuppressive molecules such as programed cell death 1 ligand (PD-L1). Furthermore, the authors discovered that non-coding Y RNA hY4 transcript was enriched in CLL-derived exosomes and acts as a ligand to facilitate the significant increase in secretion of CCL2, CCL3, CCL4, CXCL10, and IL-6 via TLR7 signaling (72).

### Macrophages

Macrophages immune cells that have important roles in antigen presentation, phagocytosis, and immunomodulation (73, 74) and their functional phenotypes are extremely versatile and dependent upon the tissue type and signals presented within it's microenvironment, thus allowing macrophages to play multiple roles in the inflammatory process (74–76). Activation of M1-phenotype macrophages leads to immunostimulation, with an increase in production of pro-inflammatory cytokines and chemokines leading to the effective elimination of pathogens and infection (77, 78), while the M2 macrophages are anti-inflammatory and promote progression of tumors, stimulate angiogenesis and wound healing (78, 79). Various studies have reported the mechanisms by which intercellular communication between cancer cells and tumor-associated macrophages, via exosomes, can regulate the function and phenotype of these immune cells.

Breast (80) and gastric (81) tumor-derived exosomes can induce a M1 pro-inflammatory response in macrophages through the activation of NFκB, which in turn stimulates production of inflammatory cytokines including GCSF, IL-6, IL-8, IL-1β, CCL2, and TNF-α. Chow et al. in 2014 further revealed that the activation of NFκB is mediated by the interaction between breast cancer-derived exosomes and macrophages, and is largely influenced by the presence of TLR2 and palmitoylated protein ligands on the surface of macrophages and tumor-derived exosomes, respectively (80). In another study, NFκB in macrophages was activated through binding of miR-21 and miR-29a, secreted by tumor-derived exosomes, to murine TLR7 and human TLR8, to trigger a TLR-mediated pro-metastatic inflammatory response to promote tumor growth and metastasis (82).

Annexin A2, which is highly expressed in breast-cancer derived exosomes, has also been reported to play a role in macrophage-mediated inflammatory response (83). Annexin A2 mediates M1 macrophage activation of p38MAPK, NFκB, and STAT3 pathways by increasing the secretion of IL-6 and TNF-α. Furthermore, priming of animals with breast cancer-derived exosomes containing high annexin A2, led to an increased level of VEGFR1 in lung and brain sections, with a concordant increase in MMP9 in tissues. Up-regulation of VEGFR1 and MMP9 expression by tumor-derived exosomes was linked with breast cancer metastasis and extracellular proteolysis and angiogenesis, respectively (83).

### Dendritic Cells

Dendritic cells are professional antigen-presenting cells (APCs) that functions to recognize, process, and present antigens to T cells of the immune system via major histocompatibility complex (MHC) molecules, along with co-stimulatory molecules and cytokines to initiate the immune system (84). Tumor-derived exosomes have been reported to be potent immune suppressors via inhibition of dendritic cell differentiation. Results from a study conducted by Yu et al. in 2007 demonstrated that administration of exosomes caused an accumulation of undifferentiated myeloid precursor cells in the spleen of mice, and *in vitro* introduction of exosomes to myeloid precursor cells resulted in the blockage of differentiation. This inhibition of dendritic cell differentiation is mediated through the induction of IL-6 by tumor-derived exosomes (85). In another study, tumor-derived exosomes was able to inhibit the differentiation of human monocyte precursors into dendritic cells in melanoma and colorectal cancers. Additionally, these monocytes gained the ability to secrete TGFβ further inhibiting the proliferation of T lymphocytes (86). Recently a study has determined that tumor-derived exosomes (TEX)-activated dendritic cells (Ta-TEXs) are able to increase the production of inflammatory mediators of dendritic cells, which include IL-6 and prostaglandin E1 (PGE1). The binding of a natural ligand, Hsp105, found on the surface membrane of tumor-derived exosomes to TLR2 and TLR4, stimulates the secretion of IL-6 and PGE1. This in turn leads to an increase in tumor cell invasion and metastasis via phosphorylation of STAT3, which in turn promotes the transcription of matrix metallopeptidase 9 (MMP9) by binding to the MMP9 promoter (87). Furthermore, pancreatic cancer-derived exosomes have been reported to down-regulate the expression of TLR4 expression in dendritic cells, via the transfer of miR-203. This led to a subsequent decrease in expression of TNF-α and IL-12 (88).

### Myeloid Derived Suppressor Cells (MDSC)

Myeloid derived suppressor cells (MDSCs) are a heterogeneous population of immature myeloid cells comprised of precursors of dendritic cells, macrophages, and granulocytes. The accumulation of MDSCs in tumor-bearing mice (89) and humans (90, 91) have been widely reported, and mounting evidence has indicated that the tumor microenvironment produces various factors that inhibit the maturation and differentiation of these immunoregulatory cells (92–94). The accumulation of MDSCs has been shown to play a role in the promotion of tumor progression, by suppressing antigen processing and presentation as well as T cell activation, which consequently inhibits immune surveillance and anti-tumor immunity (95–97).

Tumor-derived exosomes have the ability to induce the accumulation of MDSCs in tumors. Yu et al. reported that tumor-derived exosomes can be taken up by bone marrow precursor cells and induce a switch in the differentiation pathway of these myeloid cells to the MDSC pathway (85). The tumor microenvironment then increases the recruitment of immune suppression molecules PGE2 and TGF-β, into the exosomes, which then facilitates tumor growth through the induction of pro-inflammatory cytokine Cox 2. These tumor exosomes were also found to release pro-inflammatory cytokines IL-6 and tumor growth factor VEGF further enhancing its role in tumor growth (98). Exosome Hsp70 (99) and Hsp72 (100) have both been reported to expand and induce the activation of MDSCs. When treated with exosomal Hsp70 and Hsp72, MDSCs was found to significantly increase the production of pro-inflammatory cytokines including IL-6, TNF- α, VEGF, and CCL2, leading to an increase in tumor growth and metastasis (100). In accordance with these changes in phenotype, exosomal Hsp70 was also found to trigger phosphorylation of STAT3 in a TLR2-MyD88-dependent manner (101).

MyD88 is a key cytoplasmic adaptor protein required for the integration and transduction of signals generated by the TLR family (101). Recent reports have determined that MyD88 plays a critical role in the inhibition of myeloid cell differentiation into dendritic cells. Results from a study conducted by Liu et al., found that melanoma exosomes have no inhibitory effect on the differentiation of bone marrow (BM) precursor cells isolated from MyD88 knockout mice, whereas an inhibitory effect was observed with BM precursor cells isolated from wild-type mice. Furthermore, significant reduction was observed in the percentage of dendritic cells in wild-type mice exposed to melanoma exosomes, while no significant effects was observed in MyD88 knockout mice (101). This indicates that MyD88 plays a crucial role to the induction of MDSCs.

Furthermore, exosomes derived from MDSCs have been reported to promote tumor progression by facilitating the polarization of M1 macrophages with a tumoricidal phenotype, to a tumor-promoting M2 macrophage, via inhibition of macrophage production of IL-2 production (102).

### Others

As previously described, communication between cancer cells and their microenvironment can be mediated via exosomes through the transfer of their cargo which includes proteins, DNAs, messenger RNAs and microRNAs (103–105). For example, exosomes originating from arsenite-transformed hepatic epithelial cells, L-02 cells, can induce a pro-inflammatory reaction in normal liver cells by transferring exosomal miR-155 (106). Briefly, in response to arsenite exposure, NFκB will be activated to promote the overexpression of miR-155, which in turn will be transferred to normal liver cells via exosomes to influence the production of IL-6 and IL-8 to evoke a pro-inflammatory response via STAT3 (106–108). Exosomes have also been reported to secrete proteins to initiate an inflammatory response. A study has described the release of an enzyme known to be essential for protein synthesis, lysyl-tRNA synthetase (KRS), from exosomes derived from colorectal carcinoma cells (109). Caspase 8 was found to facilitate the secretion of KRS from exosomes, which in turn induced the release of cytokines and factors that are known markers for M1 and M2 macrophages, including TNF-α, C-X-C motif chemokine ligand 10 (CRG2), IL-6 and MMP9 (109). Heat shock protein, crystallin alpha B (CRYAB) has also been described to be released from glioblastoma multiforme (GBM) derived-exosomes in response to exposure to pro-inflammatory cytokines IL-1β and TNF-α, to exert an anti-apoptotic activity (110). Furthermore, results indicated that following an increase in cytokine levels in GBM cells, due to either radiation or disease, significant changes occur in the GBM derived-exosomal proteome which may promote progression of inflammation, tumor invasiveness, angiogenesis, and tumor progression (110).

## Anti-inflammatory Effects of Exosomes

Tumor inflammation plays a crucial part in the advancement of initiation, promotion, and progression of tumourigenesis through the development of a pro-tumorigenic microenvironment. This can be achieved by exosomes through the suppression of the immune system and prevention of uncontrolled inflammation. For example, exosomes can induce immunosuppression through the initiation of apoptosis in immune cells (111). In Epstein-Barr virus (EBV)-infected nasopharyngeal cells, released exosomes contain high concentrations of galectin-9 protein, which is able to induce apoptosis in mature Th1 lymphocytes (112, 113). Tumor-derived exosomes have also exhibited the ability to trigger Fas-dependent apoptosis of activated CD8^+^ T cells in both colorectal cancer as well as melanoma cells, thus contributing to tumor escape from the immune system (56, 114, 115).

### Natural Killer Cells

Natural killer (NK) cells are innate lymphoid cells that are involved in the protection of the host against infection and cancerous cells, as well as the regulation of homeostasis via destruction of activated immune cells (116, 117). Tumor-derived exosomes have been reported to suppress the activity of NK cells as a means to promote immune escape of cancer cells. These tumor-derived exosomes are able to arrest NK cell development through the release of the immunosuppressive cytokine transforming growth factor-β (TGF-β) (118). Furthermore, in a study using syngeneic BALB/c and nude mice, exosomes derived from TS/A or 4T.1 murine mammary tumor cells was able to induce tumor growth by inhibiting IL-2 mediated activation of NK cells (119). NK cell proliferation was also reported to be inhibited by exosomes produced by human breast and melanoma cell lines (119). Tumor derived exosomes have also been shown to induce Smad phosphorylation, impair cell cytotoxicity, and decrease NKG2D receptor expression, which will result in the loss of tumor cell surface markers, that would in normal situations stimulate immune response (120–122). Furthermore, exosomes are able to act as decoys by secreting NKG2D ligands, which will down-regulate NKG2D receptor-mediated cytotoxicity of NK cells (123).

### Regulatory T Cells (Treg)

Regulatory T cells (Tregs) are a dedicated subset of a larger, heterogeneous population of CD4^+^ T cells (124, 125), that has an important function in immune homeostasis, as well as a role in the regulation of various inflammatory process including autoimmunity, tissue injury, and transplant rejection (126–128). In cancer, Tregs have been found to be accumulated in the tumor microenvironment and plays a significant role in maintenance of immune tolerance via the suppression of various immune cells such as T lymphocytes (129), B lymphocytes (130), NK cells (131), dendritic cells (132), and macrophages (133). Patients with high density of Tregs in the tumor stroma have also been shown to have a worse prognosis in comparison to those with low density of Tregs (134).

Tumor derived exosomes can initiate immunosuppression through the indirect expansion and activation of Treg. This will lead to an up-regulation of Treg suppression function and enhancing Treg resistance to apoptosis (135, 136). Szajnik et al. reported that the expression of CD24^+^CD25^+^FOXP3^+^ Treg cells is significantly increased in the peripheral blood of myeloma patients in comparison to non-cancerous donors, and the serum of these patients contained high concentrations of tumor-derived exosomes. They further determined that tumor-derived exosomes was able to induce Treg expansion through mechanisms involving phosphorylation of relevant transcription factors IL-10 and TGF- β1 (136).

### Macrophages

In hepatocellular carcinoma, exosomes are able to indirectly down-regulate expression of the pro-inflammatory cytokine IL-6 as a means of suppressing the immune system. A study has demonstrated that TLR4, a fundamental signaling pathway that mediates inflammation (137, 138), was found to regulate uncontrolled inflammation through the release of exosomes via the MyD88 dependent pathway. Exosomes in turn transferred miR-let-7b to macrophages to inhibit the expression of pro-inflammatory IL-6, thus weakening tumor inflammation (139).

In another study, macrophages were found to be affected by the overexpression of miR-940 released by exosomes derived from hypoxic ovarian cancer cells, inducing an anti-inflammatory M2 polarization of macrophages to promote epithelial ovarian cancer (EOC) cell proliferation and migration (140). Another miRNA, miR-222-3p, enriched in EOC-derived exosomes, has also been revealed to increase polarization of M2 macrophages to promote angiogenesis and lymphangiogenesis in the tumor microenvironment to further encourage progression of EOC (141). Results indicated that miR-222-3p directly targets SOCS3 (suppressor of cytokine signaling 3), a negative regulator of the JAK/STAT pathway (142), which has been described to control the polarization of M1 and M2 macrophages (143–145). Suppression of SOCS3 expression was found to correlate with an increased expression of STAT3 activation (146).

A shift to M2 polarization can also be seen in macrophages exposed to exosomes released from colon cancer cells harboring gain-of-function mutant p53. These exosomes contain significantly high levels of miR-1246 which when transferred to neighboring macrophages stimulate heightened secretion of anti-inflammatory cytokines and epithelial-mesenchymal (EMT) promoting factors, thus contributing to tumourigenesis and poor prognosis (147). Another method through which exosomes mediate the polarization of M2 macrophages is by traversing the monocyte cytoplasm to induce a change in morphology and reorganization of the actin cytoskeleton. These exosomes secreted from glioblastoma-derived stem cells (GSCs) stimulate an increase of programmed death-ligand 1 (PD-L1) in monocytes, via association with STAT3, to mediate this suppressive switch (146).

In turn macrophages are also able to induce the release of exosomes to shuttle miRNAs into adjacent cells within the microenvironment. In response to activation by anti-inflammatory molecule IL-4, macrophages from breast cancer tumors has demonstrated the ability to release exosomes containing miR-233 which plays a role in tumor growth, invasion, and metastasis via direct targeting of myocyte enhancer factor (Mef2c) (148).

## Future Perspective on Exosomes in Immunotherapy

As evidenced by the numerous studies, exosomes have the ability to suppress or activate the immune system through regulation of various immune cells. Due to key features of exosomes, such as their structure, composition, and their natural ability to transport and transfer their cargo among cells, exploitation of exosomes has been acknowledged as a feasible means of immunotherapy and treatment of cancer.

Loading of immunotherapy elements such as tumor associated antigens and adjuvants into exosomes have been reported to induce strong antigen-specific immunostimulatory effects (149). For example, in one study by Morishita et al. a murine melanoma B16-BL6 tumor cell-derived exosome was engineered to carry endogenous tumor antigen and streptavidin-lactadherin, which facilitated the delivery of biotinylated CpG DNA. Delivery of these exosomes in immunized B16-BL6 tumor-bearing mice induced an *in vivo* anti-tumor effect (150). In another study by Mahmoodzadeh et al., MDA MB-231 cancer cell exosomes were loaded with staphylococcal enterotoxin B, which functions to activate T cells through the binding of MHCII. Esterogen receptor-negative (ER-) breast cancer cells treated with these exosomes were then observed to undergo significant apoptosis (151). Furthermore, studies have shown that loading of exosomes with miRNAs also has the potential to induce immune responses in their recipient cells. Momem-Heravi et al. demonstrated that B-cell-derived exosomes loaded with miR-155 was able to target macrophages *in vitro* and *in vivo* and induce the activation and differentiation of macrophages to an inflammatory M1-phenotype (152). Currently there are a number of methods to load exosomes with the desired cargo, which includes electroporation, sonication, direct transfection, and simple incubation (153). However, further understanding of the limitations and advantages of these techniques must be studied to improve the potential of exosomes as a mode of immunotherapy.

Dendritic-cell (154)-derived exosome therapy is another is another means of inducing immune response for cancer treatment. In this therapy, dendritic cells with cancer peptides are stimulated to induce the production of DC-derived exosomes that carry the desired antigens. These DC-derived exosomes are then isolated, purified, and used to induce an immune response either through presenting of specific antigens to T cells to directly activate them, or through indirect activation through the presentation of antigen-MHC complex transfer to other dendritic cells (52, 53, 155). The immune stimulatory effects of DC-derived exosomes have shown promising, but modest, results in two phase I human clinical trials in melanoma and non-small-cell lung cancer (156, 157). Subsequently, a phase II clinical trial in advanced non-small-cell lung cancer patients pre-treated with chemotherapy has shown limited efficacy of DC-derived exosomes to stimulate NK cell activation and improve the progression-free survival of patients (158).

The possibility of using exosomes as a means for cancer immunotherapy is still developing and there remain many challenges to overcome. Further knowledge must be obtained regarding the many roles that exosomes play in cancer and immunomodulation as well as the logistics for the development of exosomes as an immunotherapy. These include further research regarding techniques for effective isolation of exosomes, the possibility of off-target effects due to the transfer of unknown materials in exosomes, as well as the amount, type, and biological functions of the biomolecules to be loaded into the exosomes (159, 160).

## Conclusion

**Figure 2:**
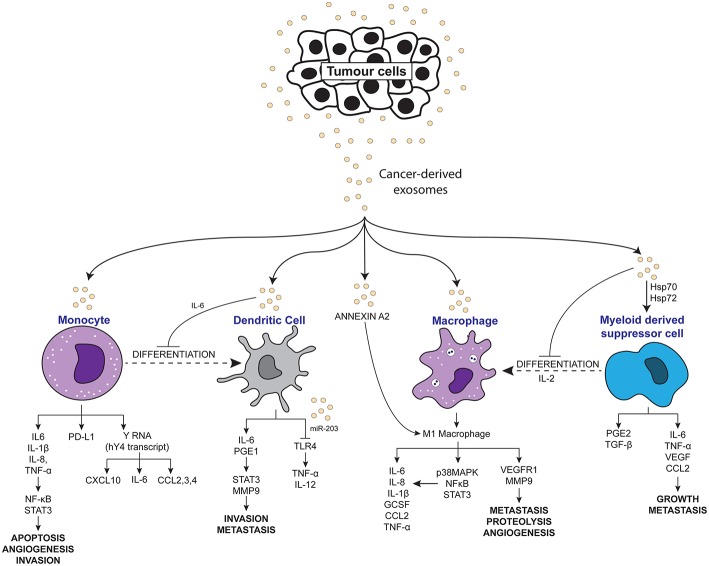
Scheme depicting the pro-inflammatory effects of tumor-derived exosomes on immune molecules.

**Figure 3:**
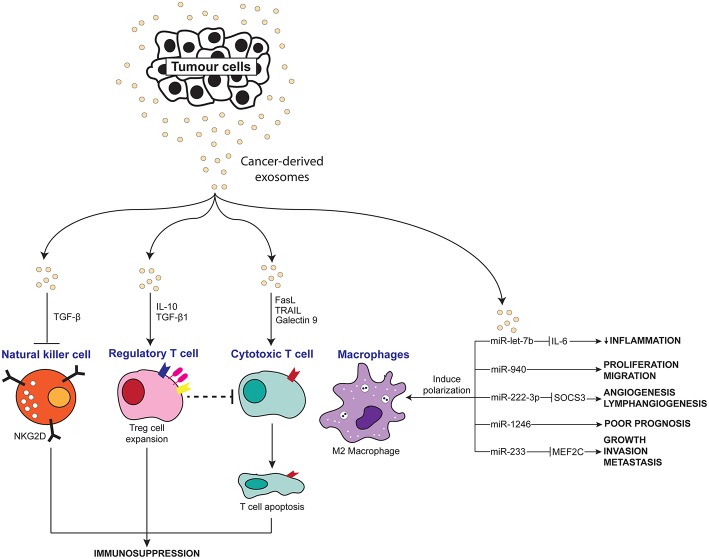
Scheme depicting the anti-inflammatory effects of tumor-derived exosomes on immune molecules.

Chronic inflammation has long been accepted to contribute to the development and pathogenesis of various cancers. Recently, many studies have explored the interaction of exosomes with the inflammatory system in promoting a pro-tumor microenvironment. As summarized in Table 1, Figures 2, 3, exosomes are able to induce pro-inflammatory and anti-inflammatory effects through the release of their cargo, which in turn modulates various cancer mechanisms including angiogenesis, metastasis, invasion, and apoptosis. Together these studies have demonstrated the importance of understanding the intricate crosstalk that exists between tumor-derived exosomes and immune molecules, as a means to acquire further understanding of the development of cancers especially in those suffering inflammatory disorders.

**Table 1:** A summary of the effects of tumor-derived exosomes on the inflammatory process.

**Immune cell**	**Exosomes derived from**	**Cargo released**	**Role in cancer development**	**References**
**PRO-INFLAMMATORY EFFECTS OF EXOSOMES**				
Monocytes	Ovarian cancer	IL-6, IL-1β, TNF- α	Increased invasion and metastasis Resistance to growth inhibition	(69)
	Chronic lymphocytic leukemia	PD-L1 Y RNA hY4 → CCL2, CCL3, CCL4, CXCL10, IL-6	Skew monocytes and macrophages toward pro-tumorigenic phenotype	(72)
Macrophages	Breast cancer	GCSF, IL-6, IL-8, IL-1β, CCL2, TNF- α	M1 macrophage activation	(80)
	Gastric cancer	GCSF, IL-6, IL-8, IL-1β, CCL2, TNF- α	M1 macrophage activation	(81)
	Lung cancer	miR-21, miR-29a	Trigger TLR-mediated pro-metastatic inflammatory response Promote tumor growth and metastasis	(82)
	Breast cancer	Annexin A2, IL-6, TNF- α	M1 macrophage activation Increased metastasis, extracellular proteolysis and angiogenesis	(83)
Dendritic cells	Breast cancer	IL-6	Inhibition of dendritic cell differentiation	(85)
	Melanoma and colorectal cancer	–	Inhibition of dendritic cell differentiation	(86)
	Melanoma and breast cancer	IL-6, PGE1	Activates dendritic cells Increase tumor cell invasion & metastasis	(87)
	Pancreatic cancer	miR-203	Down-regulates expression of TLR in dendritic cells Decrease expression of TNF- α and IL-12	(88)
MDSCs	Murine mammary adenocarcinoma	PGE2, TGF-β, Cox2, IL-6, VEGF	Expansion of myeloid-derived suppressor cells Induce tumor growth	(98)
	Renal cell carcinoma	Hsp70	Increase production of pro-inflammatory cytokines Induce tumor growth and metastasis	(99)
	Murine colon carcinoma	Hsp72	Promote MDSC suppressive functions	(100)
	Murine mammary carcinoma	–	Polarization of M1 macrophages to M2 macrophage	(102)
Others	Arsenite hepatic epithelial cells	miR-155	Increase IL-6 and IL-8 to induce pro-inflammatory response	(106)
	Colorectal carcinoma	Lysyl-tRNA synthetase (KRS)	Induce release TNF-α, CRG2, IL-6, and MMP9	(109)
	Glioblastoma multiforme	CRYAB	Induce anti-apoptosis, invasion, angiogenesis and tumor progression Promote progression of inflammation	(110)
**ANTI-INFLAMMATORY EFFECTS OF EXOSOMES**				
Natural killer cells	Acute myeloid leukemia	TGF-β	Suppress activity of NK cells Tumor escape from immune system	(118)
Natural killer cells	Murine mammary tumor cells	–	Inhibiting IL-2 mediated activation of NK cells Induce tumor growth	(119)
	Breast cancer and mesothelioma	–	Decrease NKG2D receptor expression on NK cells Suppress activity of NK cells	(120–122)
	Leukemia	NKG2D ligands	Down-regulate NKG2D receptor-mediated cytotoxicity of NK cells	(123)
Treg cells	SCCHN and oral cavity squamous cell carcinoma	–	Expansion and activation of regulatory T cells Resistance of Treg apoptosis	(136)
Macrophages	Hepatocellular carcinoma	miR-let-7b	Inhibit expression of IL-6 in macrophages Weaken tumor inflammation	(139)
	Hypoxic ovarian cancer	miR-940	Increase polarization of M2 macrophages Promote cancer cell proliferation and migration	(140)
	Epithelial ovarian cancer	miR-222-3p	Increase polarization of M2 macrophages Promote angiogenesis and lymphangiogenesis	(141)
	Glioblastoma-derived stem cells	PD-L1	Increase polarization of M2 macrophages	(146)
**ANTI-INFLAMMATORY EFFECTS OF EXOSOMES**				
	Colon cancer	miR-1246	Increase polarization of M2 macrophages Increase epithelial-mesenchymal (EMT) promoting factors Contribute to tumourigenesis and poor prognosis	(147)
	Breast cancer	miR-233	Tumor growth, invasion, and metastasis	(148)
	EBV-infected nasophyrangeal carcinoma	Galectin-9	Induce apoptosis of mature Th1 lymphocytes	(111)
Others	Squamous cell carcinoma of the head and neck (SCCHN) and melanoma	–	Fas-dependent apoptosis of activated CD8^+^ T cells Tumor escape from immune system	(114)
	Colorectal	–	Fas-dependent apoptosis of activated CD8^+^ T cells Tumor escape from immune system	(115)

## Author Contributions

NO drafted the manuscript. NA conceived the idea. NA and RJ provided critical feedback.

### Conflict of Interest Statement

The authors declare that the research was conducted in the absence of any commercial or financial relationships that could be construed as a potential conflict of interest.
